# Therapeutic Implications of Black Seed and Its Constituent Thymoquinone in the Prevention of Cancer through Inactivation and Activation of Molecular Pathways

**DOI:** 10.1155/2014/724658

**Published:** 2014-05-18

**Authors:** Arshad H. Rahmani, Mohammad A. Alzohairy, Masood A. Khan, Salah M. Aly

**Affiliations:** ^1^Department of Medical Laboratories, College of Applied Medical Sciences, Qassim University, Buraydah, Saudi Arabia; ^2^Basic Health Science, College of Applied Medical Sciences, Qassim University, Buraydah, Saudi Arabia; ^3^Department of Pathology, Faculty of Veterinary Medicine, Suez Canal University, Ismailia, Egypt

## Abstract

The cancer is probably the most dreaded disease in both men and women and also major health problem worldwide. Despite its high prevalence, the exact molecular mechanisms of the development and progression are not fully understood. The current chemotherapy/radiotherapy regime used to treat cancer shows adverse side effect and may alter gene functions. Natural products are generally safe, effective, and less expensive substitutes of anticancer chemotherapeutics. Based on previous studies of their potential therapeutic uses, *Nigella sativa* and its constituents may be proved as good therapeutic options in the prevention of cancer. Black seeds are used as staple food in the Middle Eastern Countries for thousands of years and also in the treatment of diseases. Earlier studies have shown that *N. sativa* and its constituent thymoquinone (TQ) have important roles in the prevention and treatment of cancer by modulating cell signaling pathways. In this review, we summarize the role of *N. sativa* and its constituents TQ in the prevention of cancer through the activation or inactivation of molecular cell signaling pathways.

## 1. Introduction

**Figure 1:**
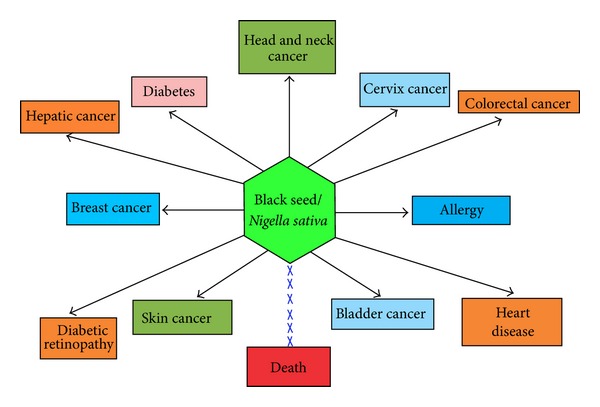
*N. sativa* shows an important role in the management of various diseases.

The cancer is probably the most dreaded disease of mankind and a major health problem worldwide. The exact cause of cancer development and progression is not completely known, but it is considered to be due to alteration of genetic and epigenetic pathways. Altered function/expression of various genes such as tumor suppressor genes, apoptotic genes, oncogene, and other genes has been noticed in various tumors [1–7]. Current modes of treatment based on synthetic drugs/chemotherapy have limited potential, because they are toxic and expensive and also alter the functioning of the cell signaling pathways. Natural drugs which are safe, affordable, and effective are needed to control the cancer development and progression. Natural products have been used for thousands of years in the management of several diseases including various types of cancer. They have therapeutic implications in various cancers via inhibiting angiogenesis process and activation of tumor suppressor gene and also show an important role in the modulation of other several activities. Earlier reports have shown that diet rich in fruits, vegetables, cereal grains, and spices decreases the risk of cancer growth and its metastasis [8–10]. Most of the drugs in chemotherapy act as monotarget molecules, whereas medicinal plants have multitarget molecules that can regulate the cancer growth and its progression.* Nigella sativa* has many beneficial constituents which can be used in the treatment of various diseases (Figure 1).* Nigella sativa* is commonly used medicinal plant which has been used for thousands of years in traditional system of medicine for example, Ayurveda, Unani, Arabic and Chinese medicine. It is claim that Prophet Muhammad (PBUH) said “Use Black seed regularly, since it is a cure for every disease except death” [11].

It is also known that herbal constituents may play an important role in cancer cure through antitumor activity or by suppressing bioactivation of carcinogen [12]. There are numerous medicinal plants/products which are beneficial for health and also have antitumor, antimicrobial, antibacterial, and antioxidant nature [13–19]. An important study summarized the importance of black seed oil and its active compound TQ in the diseases management including cancer [20]. TQ is considered as potent anticarcinogenic and antimutagenic agent [21, 22] and aqueous and alcohol extracts of* N. sativa* were found to be effective* in vitro* in inactivating MCF-7 breast cancer cells [23]. In this review, we summarize the role of* N. sativa* and its constituent TQ in the prevention of cancer through activation or inactivation of various genetic pathways.

## 2. Active Ingredients and Chemical Structure of* N. sativa*

**Figure 2:**
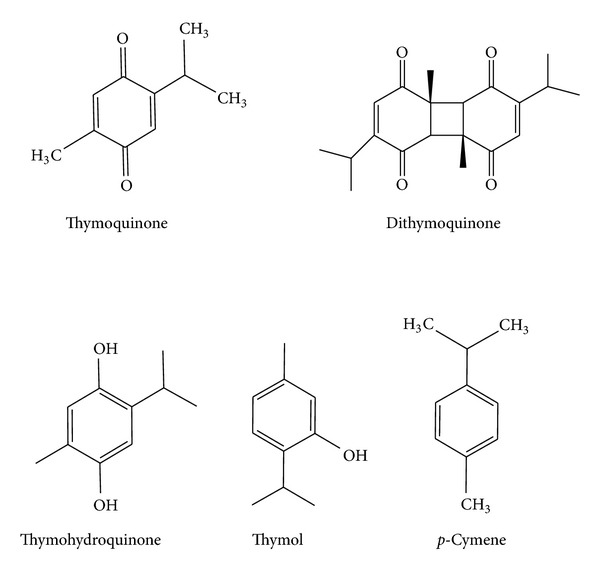
Chemical structure of active ingredients of* Nigella sativa*.

Numerous active compounds were isolated and identified in* N. sativa* (Figure 2).* N. sativa* and its constituents showed therapeutic effects by modulating various cell signaling activities. Chief constituents/ingredients of* N. sativa* are thymoquinone (TQ), dithymoquinone (DTQ), thymohydroquinone (THQ), and thymol (THY) [24]; p-cymene, 4-terpineol, and t-anethol.* N. sativa* seeds contain other ingredients as well, such as carbohydrates, fats, vitamins, mineral elements, proteins, and essential amino acids [24–28]. Black seed also contain nigellidine,nigellimine,nigellcine [29], saponine and water soluble triterpene [25].

## 3. Mechanism of Action of Thymoquinone in Cancer Prevention

**Figure 3:**
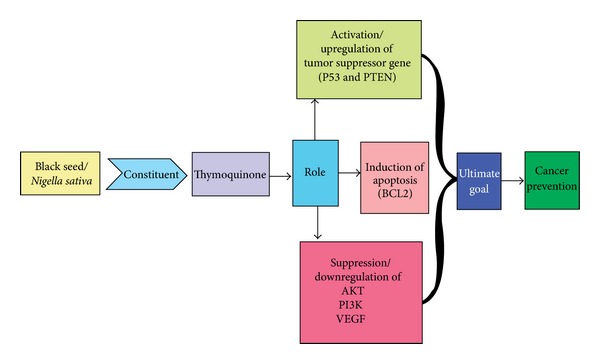
*N. sativa* shows a vital effect in the prevention of cancer through upregulation of tumor suppressor gene and inhibition of VEGF, Akt/PI3K pathways.

Cancer is a multifactorial disease, including alteration in genetic pathways [30]. Numerous studies have shown that TQ has therapeutic potential in the health management/human health as well as in the prevention of cancer via modulation of genetic cascades. TQ shows a critical role in controlling cancer via the activation of tumor suppressor gene, phase II gene/enzymes, and peroxisome proliferator-activated receptors (PPARs), inactivation of angiogenesis and anti-inflammatory gene, and induction of apoptosis. An important finding in this regard proved that TQ showed anticancer effects and regulated apoptosis in doxorubicin-resistant human breast cancer cells (MCF-7/DOX cells) [31]. That TQ induces apoptosis in doxorubicin-resistant breast cancer cells via upregulation of PTEN at the transcription level, and upregulated PTEN plays a significant task in inhibition of phosphatidylinositol-3 kinase/Akt pathway and induces p53 and p21 protein expression [31]. TQ also modulates various pathways such as inhibition of phase I enzymes (cytochrome p450) and activation of phase 2 detoxifying enzymes (glutathione transferase and N-acetyl transferase), inhibition of multiple signal transduction pathways that trigger cell proliferation, and invasion and activation of PPARs. TQ inhibits NF-*κ*B activation with resultant downregulation of multiple inflammatory genes. A recent study reported that TQ intervenes with TNF and NF-kappa-B signaling during TQ-mediated induction of apoptosis in cancer cells [32]. TQ finally acts as multiple target modulator in the cancer control via modulation of genetic pathways (Figure 3).

## 4. The Implications of* N. sativa* in Cancer Therapy

Several active constituents are present in* N. sativa and these constituents play a role in tumor prevention*. The seeds contain both fixed and essential oils, proteins, alkaloids, and saponin [33]. Earlier studies have reported the precise mechanism of tumor inhibition by* N. sativa* volatile oil. For instance, thymoquinone was found to have antioxidant effects in animal models [34–36]. Black seed and its chief constituents TQ shows therapeutics role in diseases control including cancers such as pancreatic, osteosarcoma, bladder, breast, colon, skin and lung and other diseases [37–44]. Earlier investigators showed that oral administration of TQ was effective in increasing the activities of quinine reductase and glutathione transferase and suggested that TQ may be a useful drug in treating chemical carcinogenesis and toxicity in liver cancer [45]. An important study showed that black seed preparations have a cancer chemopreventive ability and also reduce/suppress the toxicity of standard antineoplastic drugs [46]. Another experimental study reported that black seed extract plays an important role in the prevention of skin carcinogenesis via inhibition of the two-stage initiation-promotion [47]. TQ shows various biological activities via modulation of antineoplastic, antioxidant, antitumor, and antimicrobial effects [48–52]. TQ plays a role in the prevention of cancer via modulation of various genes including tumor suppressor genes, apoptotic genes, hormonal receptor genes, and detoxifying genes. The study proposed the detailed mechanism of action of genes in the management of cancer is described as the following.

### 4.1. Effect of Thymoquinone on Tumor Suppressor Gene

Mutation or alteration of tumor suppressor genes contributes to the development of cancer by inactivating the inhibitory functions.* N. sativa *and its constituents play a role in the prevention of cancer via the activation of tumor suppressor gene. PTEN is a multifunctional phosphatase, whose major substrate is phosphatidylinositol-3,4,5-trisphosphate (PIP3) [53] and lipid phospahatse activity of pten play an important role in dephosphorylation of PIP3 and also inhibit the Akt/PI3K pathways. Altered expression of PTEN has been noticed in cancer [54]. However, PI3K/Akt inactivation and upregulation of PTEN are of prime importance in the cancer prevention. An important study showed time-dependent increase of PTEN in MCF-7/DOX cells and it was observed that TQ treatment induced an increase in PTEN mRNA by 1.8-, 2.0-, 3.8-, 5.9-, and 7.9-fold after 1, 2, 4, 8, and 24 hours, respectively [31]. TQ also plays a significant role in the modulation of other tumor suppressor genes such as p53, P21, and p27.

p53 is considered as guardian of genes and the function of p53 is altered in approximately 50% of cancers. Studies in the favor of TQ showed that TQ plays role in the modulation of p53 and finally suppresses the tumor development and progression. Earlier studies showed that TQ induces apoptosis through p53-dependent pathways in human colon cancer cells and animal models [55, 56] and apoptosis induction by TQ was associated with 2.5–4.5-fold increase in mRNA expression of p53 and the downstream p53 target gene, p21WAF1 [55].

P21WAF1/CIP1 is a cyclin-dependent kinase (cdk) inhibitor and is one of the key mediators of p53-dependent cell cycle arrest after DNA damage [57, 58]. A finding showed that TQ induces apoptosis via upregulation of PTEN at transcription level in doxorubicin-resistant breast cancer cells. Furthermore, upregulated PTEN inhibited the PI3K/Akt pathway and induced p53 and p21 protein expression [31]. In p53-null myeloplastic leukemia HL-60 cells, thymoquinone induced apoptosis by activating caspase-3, caspase-8, and caspase-9 [59].

### 4.2. Regulation of Apoptosis and Necrosis by TQ

Any modifications/changes which occur in the normal process of apoptosis may increase cell survival and promote tumor development and progression [60, 61]. Earlier investigators have shown that plants (*Glycyrrhiza uralensis* Fisch) may have chemopreventive effects against different types of cancer by modulating the expression of the Bcl-2/Bax apoptotic regulatory factors [62]. Another study showed* Myristica fragrans* Houtt induced apoptosis of Jurkat leukemia T cell line in mechanisms involving SIRT1 mRNA downregulation [63].

*N. sativa* plays a major role in anticipation of cancer via the induction of proapoptotic factors Bak/Bax or downregulation of antiapoptotic proteins Bcl-2 and Bcl-xL that cause the activation of caspase in cancer cell without alteration in normal cells.* Nigella sativa* and its constituents TQ showed vital effects as anticancer and also trigger apoptotic cell death in human colorectal cancer via a p53-dependent mechanism [55, 64]. A study demonstrate that Thymoquinone(TQ) treatment shows the down-regulation of constitutive activation of AKT via generation of reactive oxygen species (ROS) and it also causes conformational changes in Bax protein [65]. TQ also causes apoptosis in HEp-2 human laryngeal carcinoma cells through activation of caspase-3 and inhibitors of caspases significantly reduced the TQ-induced apoptosis [66]. This finding shows that TQ plays a role in the induction of apoptosis and also has a significant role in the regulation of the caspase pathways. A study has shown that TQ inhibits G1 phase of cell cycle by increasing the expression of the cyclin-dependent kinase inhibitor p16 and downregulating the cyclin D1 protein expression in papilloma cells [67].

Another important study showed that all anticancer-active derivatives of Thymoquinone shows a role in induction of apoptosis associated with DNA laddering, a decrease in mitochondrial membrane potential and also increase in reactive oxygen species [68]. TQ also exhibits the anticancer activity through the modulation of multiple molecular targets, including p53, p73, PTEN, STAT3, PPAR-*γ*, activation of caspases, and generation of ROS [69].

Necrosis appears to be the result of acute cellular dysfunction in response to severe stress condition or after exposure to toxic agents [70]. Oxidative stress is one of the most important factors that play a role in various types of pathogenesis. Our body has defense mechanism against adverse effects of free radicals. If any imbalance occurs between free radical production and antioxidative defense system, that cause damage to macromolecules (DNA and protein). The constituents from plants play an important role in neutralisation of the oxidative stress via antioxidant properties. TQ oral administration showed protective role in various organs against oxidative damage induced by free radical generating agents [71]. Furthermore, TQ constitutes strong antioxidant properties via the scavenging ability of different free radicals, its scavenging power being as effective as SOD against superoxide anions [72, 73]. Earlier report showed that TQ inhibited the expression of the inducible nitric oxide synthase (iNOS) and iNOS mRNA expression in rat lipopolysaccharide stimulated peritoneal macrophage cells [74]. TQ shows an important role in suppression of tumor necrosis factor. An important study in human chronic myeloid leukemia cells KBM-5 showed that thymoquinone (TQ) suppressed tumor necrosis factor-induced NF-*κ*B activation in a dose- and time-dependent manner and also played a role in the inhibition of activation of NF-*κ*B that was induced by several carcinogens and inflammatory stimuli [75].

### 4.3. Effect of TQ on Angiogenesis

Angiogenesis is a complex process, which plays a role in various types of cancer [76–79]. TQ inhibits angiogenesis by suppressing the activation of one of the important motifs of angiogenesis. Endothelial cell migration shows a critical effect in the angiogenesis and TQ inhibited HUVEC migration in a concentration-dependent manner [80].

An experimental study showed that serum levels of VEGF were significantly low in TQ-treated group compared to control group. However, this result indicates that TQ has potential or ability to suppress the VEGF productions and prevents the tumor growth through antitumor angiogenesis [81]. A finding on osteosarcoma showed antitumor and antiangiogenesis effects of TQ through the NF-*κ*B pathway [82]. An important finding suggests that TQ increases the antiangiogenic activity by inhibiting HUVEC differentiation into tube-like structures [83].

Another study of TQ in the angiogenesis on human pancreatic cancer cell line PANC-1 showed that incubation of EPCs with TQ decreased the tube-forming capacity [84].

### 4.4. TQ-Induced Inhibition of COX

Cyclooxygenase (COX) is also known as prostaglandin (PG) H synthase and catalyses the stages of prostanoids synthesis [85]. There are two types of COX enzymes, COX1 and COX2. COX1 is expressed in almost all tissue is the inducible isoform, which is regulated by growth factors and cytokines [86] and is overexpressed in inflammatory conditions. The overexpression of COX2 was observed in a wide range of cancers such as lung, stomach, breast, and pancreatic cancer [87–90]. The overexpression of COX2 plays a vital role in the upregulation of angiogenesis by prostaglandin and also shows effect in apoptosis by increasing the resistance to apoptosis [91]. COX2 also shows the inhibition of clinical behavior of some tumors [92]. However, COX2 inhibition is a critical step in the prevention of cancer. The inhibitor of COX2 shows a significant effect on the inhibition of COX2 action and also shows side effect in the tissues. Therefore, natural product is good choice in the prevention of tumor development through the inhibition of COX2. TQ showed a critical effect on the inhibition of COX2 expression and prostaglandin production in a mouse model of allergic airway inflammation [93].

### 4.5. Effect of TQ on Nuclear Factor-*κ*B (NF-*κ*B)

NF-*κ*B is a transcription factor belonging to the Rel family of proteins that are involved in regulation of various genes [94, 95]. There are numerous carcinogens playing a role in the activation of NF-*κ*B [96–100]. However, the regulation of NF-*κ*B action is a key step towards the prevention of cancer development and progression. Although the exact mechanism of action of TQ is not fully understood, it might be due to TQ inducing oxygen reactive species through the PI3K/Akt and p38 kinase pathways [101]. The activation of NF-*κ*B has been noticed in various tumors [102–104]. A recent study reported that TQ intervenes in NF-kappa-B signaling during TQ-mediated induction of apoptosis in cancer cells [32]. Another report showed that TQ has strong anti-inflammatory activity in several PDA cell lines and demonstrates that TQ mediates its effects by reducing the activity and transcription of NF-*κ*B [105]. Another finding* in vitro* showed that TQ inhibits tumor angiogenesis and tumor growth by suppressing NF-*κ*B and its downstream molecules [82]. TQ has been shown to have antitumor effects on bladder cancer in both* in vitro* and* in vivo* models through the downregulations of NF-*κ*B and its regulated molecules such as XIAP [106]. Recent studies reported that TQ significantly downregulates NF-*κ*B and MMP-9 in Panc-1 cells [107].

### 4.6. Effect of TQ on Androgen Receptor (AR)

Androgen plays a crucial role in the growth, differentiation, and maintenance of prostate tissue and also shows the effect in prostate cancer [108]. Earlier investigators showed that Casodex (bicalutamide), a specific inhibitor of AR, blocks the ability of G_1_ phase of AR-positive LNCaP prostate cancer cells to enter S phase [109–111].

Cytotoxic chemotherapy and radiotherapy are not very effective in survival benefit for patients with hormone-refractory prostate cancer. Although taxane drugs are helpful, they are not fully effective [112–115]. The presently used chemotherapy shows adverse effects and also alters the normal mechanism of action of genes.* N. sativa* is a good candidate for chemoprevention that inhibits AR signaling. An recent study on animal model showed that thymoquinone suppresses the expression of AR and E2F-1 necessary for proliferation and viability of androgen-sensitive as well as androgen-independent prostate cancer cells both* in vitro* and* in vivo* and, interestingly, produced no clear side effects [116].

### 4.7. Effect of TQ on Lipoxygenase Activity

The lipoxygenase is a family of nonheme iron-containing dioxygenases that catalyze the stereospecific oxygenation of the 5-, 12-, or 15-carbon atoms of arachidonic acid [117–119]. The metabolism of arachidonic acid by the lipoxygenase (LOX) pathway generates eicosanoids and these factors show a critical role in the pathogenesis of different human diseases including cancer [120]. Earlier investigators have observed the upregulation of LOX in various types of cancers [121, 122]. The inhibition of LOX in the prevention of cancer is one of the critical steps towards management of cancer. An important report suggested that COX2 may play an important role in pancreatic tumour development and therefore be a promising chemotherapeutic target for the treatment of pancreatic cancer [123].* N. sativa* plays a role in the downregulation of LOX or inhibition of 5-lipoxygenase and leukotriene C4 synthase in human blood cells by TQ [124].

### 4.8. Effect of TQ on Signal Transducers and Activators of Transcription

Signal transducers and activators of transcription 3 (STAT3) are the members of the STAT family and play a role in the regulation of transcription [125]. Signal transducers and activators of transcription (STAT) are the part of the signal transduction pathway of many growth factors, cytokines and are activated by phosphorylation of tyrosine and serine residues by upstream kinases [126]. Numerous studies showed that TQ suppresses STAT-3 phosphorylation and the expression of its downstream signalling effectors Bcl-2, Bcl-XL, cyclin D1, survivin, Mcl-1, and VEGF [127, 128].

### 4.9. Effect of TQ in Phase I and II Enzyme/Genes

Xenobiotics are chemicals in our environment and exposure to these chemicals causes cancer. When these chemicals enter into our body, the body metabolizes them via phase I (CYP450) and phase II (GST) gene/enzyme.

**Figure 4:**
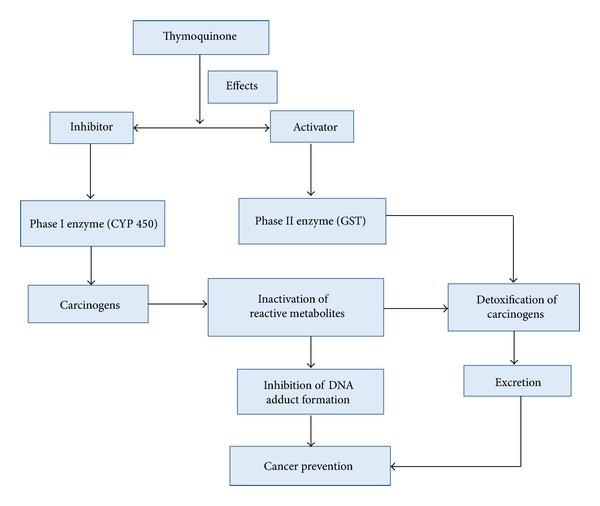
Thymoquinone shows a vital role in prevention of cancer via modulation of phase I and phase II enzymes.

Cytochrome P450 (CYP) plays a role in the activation of carcinogens and glutathione S-transferase (GST) shows the effect in the deactivation of reactive metabolites/carcinogens. However, the regulation of theses enzymes is important in the prevention of cancer. Black seeds and their constituents show a vital role in the control of cancer via inactivation and activation of phase I and II genes (Figure 4). An important study showed that TQ significantly inhibited CYP2D6 and CYP3A4 mediated metabolism of DEX in human liver microsomes [129].

Oral administration of TQ is a promising prophylactic agent against chemical carcinogenesis and toxicity in liver tissues by increasing the activities of quinone reductase and glutathione transferase [130].

### 4.10. Effect of TQ in Peroxisome Proliferator-Activated Receptors (PPARs) Activation

Peroxisome proliferator-activated receptors (PPARs) are ligand-activated transcription factors [131, 132]. PPAR plays a key role in tumor suppression and also induced apoptosis as well as antiproliferative effect in various cancer cell lines [133–138]. The upregulation of PPAR plays a role in the prevention of cancer by regulating various genetic pathways including apoptosis. Presently PPAR-*γ* agonist is in use for the activation, but these agonists show the side effect. Natural products are the best remedy, as they are safe without any side effect. TQ plays a significant role in the regulation of PPAR-*γ* without any alteration in tissues. A study of TQ activity showed that it acts as antitumor by modulation of PPAR-*γ* activation [139].

## 5. Toxicity of TQ

Drugs used in the treatment of diseases were considered to be safe in doses pattern. Medicinal plants have important value in the health management because of their low toxicity. Numerous studies have been investigated to know the toxicological properties of TQ* in vitro* and* in vivo* [140–142]. The oral and intraperitoneal lethal dose (LD50) in animal models such as rats and mice has been successfully assigned by earlier researcher [143, 144]. The safety, tolerability, and less toxicity of* N. sativa* at higher doses are established via human clinical trials. A study showed that TQ at the concentrations of 0 to 10**μ**M was not cytotoxic to fibroblast-like synoviocytes [145]. Earlier study showed that* Nigella sativa* did not show any toxicity effect on liver and supplementations of* Nigella sativa* reduce the alanine aminotransferase (ALT) level and aspartate aminotransferase (AST) level treated rats compared to the control doses of rats [146]. Another study reported that 0.6 mg/kg/day oral dose of TQ is suitable for humans [145] and 0.05 mg/kg/day of* Nigella sativa* extract for postmenopausal women [147]. Another report showed that the LD50 of oral administration of TQ was 2.4 gm/kg (1.52–3.77, 95% CL). Numerous studies in the support of* N. sativa* have shown that no evidence of* N. sativa* fixed oil toxicity was observed, when administered in different doses up to 10 mL/kg body wt., p.o. [148].* N. sativa and *its seed powder did not show any toxic effects and were safe at very high doses (28 gm/kg orally) and its oil was also safe at dose (28.8 ml/kg) in rabbits and rats respectively [149, 150].

## 6. Role of Analogues of TQ in Cancer

Several types of TQ derivatives have been synthesised and successfully tested in animal models with better efficiency. An important study in this prospective has synthesized twenty-seven analogues of TQ by the modification at carbonyl and benzenoid sites and test was performed to check the biological activity against pancreatic cancer cell lines. Analogues such as TQ-2G, TQ-4A1, and TQ-5A1 were found to be more effective than parental TQ in terms of inhibition of cell growth, induction of apoptosis, and modulation of transcription factor, NF-*κ*B [151]. An earlier study has conjugated TQ with fatty acids to enhance the membrane penetration capacity and antitumor activity [69, 152] and also terpene conjugated with TQ showed greater antitumor activities than the parental drug [153]. The triterpene betulinic acid conjugate of TQ also exhibits up to 200-fold better activity in HL-60 leukemia cells as compared to the control [153].

A recent study showed that Poloxin, a synthetic derivative of thymoquinone play a role in the induction of mitotic arrest and prolongs the mitotic duration, accompanied by Plk1's (member of Polo-like kinases) mislocalization at kinetochores and centrosomes with reduced *γ*-tubulin [154]. Antiproliferative effect of various thymoquinone analogues was tested with six human cell lines and the lead compound, ON01910, blocked the cancer cells and, furthermore, molecule OC2-23 was ten times more effective than the lead compound, ON01910 [155]. The other derivatives of thymoquinone such as 4-acylhydrazones and 6-Alkyl were also tested for growth inhibition in human HL-60 leukemia, 518A2 melanoma, KB-V1/Vb1 cervix, and MCF-7/Topo breast carcinoma cells. Among them, 6-hencosahexaenyl conjugate showed the most active effect with IC50 values as low as 30 nmole/mL in MCF-7/Topo cells [156].

## 7. Clinical Trials Based Study of* N. sativa*

Diseases are caused due to multifactorial alteration, including changes in hormone receptor gene, angiogenesis process, tumor suppressor gene, and apoptosis cascade and metabolic process. Current modes of treatment based on synthetic drugs are effective; unfortunately they show the limitation as monotarget capability and adverse side effect. The drugs with multitargets potentiality are needed to control the diseases by modulating various genetic pathways*. N. sativa* shows therapeutic role in the management of diseases via multiple target activities in the clinical base study. Earlier investigators have shown that* N. sativa* and its constituents show vital effect in disease control without any toxic effect. A few studies have been performed on human to check the efficacy of TQ in disease management. The first study of TQ in human trial of advanced malignant cancer patients showed that treatment with TQ tolerated the drug at oral doses up to 2600 mg/day [157]. A study conducted a randomized, double-blind, and placebo-controlled trial to check the efficacy of oral* N. sativa *(NS) seed extract supplement in patients with mild hypertension (HT) and results suggest that the daily use of NS seed extract for 2 months may have a blood pressure-lowering effect in patients with mild HT [158]. Another report showed that* N. sativa* powder administered to the hypercholesterolemia patients for two months was found to reduce the total cholesterol and triglycerides [159].

As per clinical trials based study on cancer patients, a few studies have been performed to check the efficacy of black seed and their constituents in cancer. The experimental study of animal model showed that dose and efficacy of TQ in disease management are with no toxic effect. Clinical trial based study is needed to confirm the efficacy in cancer control through multitargets activity of various genes.

## 8. Conclusions

Cancer is a major health problem worldwide and also a notorious killer disease in both men and women. Chemotherapy/radiotherapy is the current mode of treatment that exhibits adverse side effect.* N. sativa* may constitute a good therapeutic approach in the prevention of cancer through regulation of molecular process. This review highlights the therapeutic role of* N. sativa* and its constituent TQ and outlines their mechanism in the prevention of cancer through the inactivation or activation of multiple molecular pathways. The molecular features of* N. sativa* combined with other properties are the mainstay in the management of cancer.
